# Core Intuitions of Psychological Non-Contradiction: Infants Assume That Individual Agents Act and Communicate Coherently

**DOI:** 10.1162/OPMI.a.339

**Published:** 2026-03-15

**Authors:** Olivier Mascaro, Ágnes Kovács

**Affiliations:** Integrative Neuroscience and Cognition Center, Université Paris Cité, CNRS, Paris, France; Department of Cognitive Science, Central European University, Vienna, Austria

**Keywords:** infancy research, psychological coherence, reasoning, communication, social cognition, contradiction, goal attribution, resolving incoherence

## Abstract

Humans generally posit that contrary mental states are unlikely to co-exist within a single mind. We tested the early ontogeny of this assumption in two domains: action and communication. Studies 1A and 1B tested whether 9-month-old infants assume that agents act coherently. Infants watched interactions between two hands whose owner(s) were invisible. In the contrary goals condition, the hand performed contrary actions—one hand reached for an object while the other impeded it. Later, during test trials, infants learned that the hands belonged to one or two people. Looking-time patterns across the contrary goals and a baseline conditions indicated that clear goal conflict led infants to infer two agents, suggesting they viewed it as unlikely for a single person to thwart their own goal. Study 2 tested whether infants assume communicative coherence, testing whether they assume that a single informant is unlikely to entertain and communicate conflicting information while two informants might do so. Informants pointed to indicate a toy’s location to 15-month-olds. When two different informants each pointed to a different place, infants did not follow one pointing gesture more than the other. However, when a single informant pointed successively to two locations, infants followed the second gesture, implying they viewed it as an updated, not contradictory, message. Thus, infants assumed that a single informant is unlikely to contradict themselves (i.e., by asserting that a toy is simultaneously in two locations). These findings reveal an early-emerging assumption of psychological coherence in infants’ representation of other minds, across both action and communication contexts.

## INTRODUCTION

A large share of research on representations of the mind has focused on attributions of a single mental state, such as a goal or belief, to agents (Gergely et al., 1995; Kovács et al., 2010; Onishi & Baillargeon, 2005; Premack & Woodruff, 1978; Wimmer & Perner, 1983; Woodward, 1998). Crucially, each “mind” contains a plethora of mental states that are organized in a structured fashion. Therefore, mentalizing should also be sensitive to the systematic relationships that exist between mental states within a mind. Among these relationships, the assumption of coherence is likely to play a crucial role. An agent is unlikely to aim at achieving a goal and, at the same time, at preventing themselves from achieving the same goal. Similarly, an agent is unlikely to believe that something is the case and is not the case at the same time. Thus, a principle of psychological coherence regulates the relations between mental states within the mind.

### The Principle of Psychological Coherence

The view of psychological coherence as a guiding principle of mental state representations can be traced back to Aristotle’s formulation of the law of non-contradiction (Lukasiewicz & Wedin, 1971). In Aristotle’s (350 BC/2020, Book IV, Part 3) terms, “it is impossible for anyone to believe the same thing to be and not to be”. A few thousand years later, social and cognitive sciences have shown that Aristotle was partly wrong. People at times hold contrary mental states. This happens, for example, when conspiracy theorists’ strong belief that officials are engaged in a cover-up leads them to endorse mutually incompatible viewpoints: the more they believe that Princess Diana faked her death, the more they believe that she was murdered (Wood et al., 2012). Similarly, there are cases in which an agent may form action plans or integrate sets of preferences in ways that are partly inconsistent (Tversky, 1969). In short, people are not always entirely coherent in their thoughts or actions.

Yet, assumptions of psychological coherence are likely to guide the interpretation of agents’ actions for at least two reasons. First, if we grant that cognitive systems function generally well, they should form relatively accurate representations of the world and relatively efficient action plans. For this reason, it seems reasonable to assume that people rarely hold inconsistent sets of beliefs or goals (Thagard, 2002). Second, assumptions of coherence play a central role in our representation of others’ mental states. Coherence is a central tenet of the assumption of rationality guiding humans’ interpretation of the behaviors of agents, both when attributing beliefs (Dennett, 1987; Millikan, 2006), or action plans (Bratman, 1983). For this reason, our capacity to predict and interpret the behavior of others breaks down when we attribute mutually incompatible mental states to an agent. For example, if one accepts that an agent can aim for both “p” and “not-p” at the same time, it is hard to predict what the agent will do.

The principle of psychological coherence applies to a single mind: two agents can hold mutually incompatible beliefs or goals, but a single agent is unlikely to do so. Moreover, it is relative to a particular state of affairs: one agent can change their mind (first aiming for “p” and then for “not-p”) or update their beliefs at a consecutive point in time, but one agent is unlikely to aim for both “p” and “not-p” or to believe both “p” and “not-p” at the same time. In this paper, we focused on the early ontogeny of coherence assumptions applied over actions. We assessed whether infants assume that agents act coherently when performing simple instrumental and communicative actions.

### Assumptions About Coherence in Instrumental Actions

Studies 1A and 1B investigated whether infants assume that individual agents act in a coherent manner. Very often, applying this assumption requires processing conflicts between goals, i.e., situations in which someone’s actions thwart the achievement of a goal. Infants seem to detect and process such conflicts early. They have been reported to prefer characters who help others achieve goals rather than those who obstruct them (Hamlin et al., 2007, 2011; however, for debates about replicability and interpretation, see Lucca et al., 2025; Powell & Spelke, 2018, experiment 4; Salvadori et al., 2015; Schlingloff et al., 2020). Moreover, infants infer social dominance by observing which agent prevails in goal conflicts (Bas & Sebastian-Galles, 2021; Enright et al., 2017; Gazes et al., 2017; Mascaro & Csibra, 2012, 2014; Mascaro et al., 2023). They expect dominant agents—identified by cues such as size or number of allies—to consistently prevail when their goals conflict with those of subordinates (Meng et al., 2019, 2021; Pun et al., 2016, 2022; Thomsen et al., 2011). Infants and toddlers also evaluate agents differently depending on whether they succeed or defer in goal conflicts (Thomas & Sarnecka, 2019; Thomas et al., 2018). Thus, infants detect situations in which two agents’ goals are conflicting, and can track their outcome.

Studies 1A and 1B capitalize on these results to investigate infants’ ability to apply assumptions of coherence over an individual’s actions. A rational agent is unlikely to prevent themselves from achieving their goals for two reasons. First, they are unlikely to aim for mutually incompatible end-states (e.g., “p,” and “not-p”) at the same time. Second, a rational agent is likely to select actions that will support rather than hinder the achievement of their goals (Bratman, 2009). These assumptions are not just central to predicting and interpreting others’ behaviors. They also allow us to set boundaries between minds. Several agents often hold contrary beliefs (e.g., when they disagree) or goals (e.g., when they compete). Meanwhile, a single agent is unlikely to disagree or compete with herself, or at least when an action plan is initiated, we assume that one of the potentially competing goals prevails and the other is overridden (e.g., when the desire to eat healthy food prevails over the crave for sweets).

Therefore, evidence of conflicting beliefs or goals resulting in conflicting action plans gives the impression that several distinct minds cohabit within a single physical entity. Indeed, fiction is rife with stories in which conflicting mental states give the impression that two minds coexist and conflict within a single body. For example, the hand of Kubrick’s Doctor Strangelove, at times trying to clutch his own throat while the doctor tries to control it with his other hand, seems to be driven by a will of its own. The illusion that conflicting goals imply the existence of distinct minds is so powerful that in actual cases of alien hand syndrome, in which a person experiences their limbs acting independently from themselves, patients often say another agent controls their own limb and refer to it in the third person (Biran & Chatterjee, 2004). Therefore, we propose that assumptions of psychological coherence are intuitively used to set boundaries between minds, and such assumptions might be present early on. Studies 1A and 1B assessed this hypothesis in 9-months-old infants. We tested whether they assume that a single agent is unlikely to prevent themselves from achieving their goals, while it may not be surprising if one agent prevents the goal of another agent. In short, we tested infants’ sensitivity to how psychological coherence shapes actions.

## STUDY 1A

### Operationalization Principle

Study 1A tested whether infants assume that a single person is unlikely to prevent themselves from achieving their goals. To this end, 9-month-olds were familiarized with a scene in which two hands were visible while their owners were hidden behind a screen. The screen disappeared by sliding upwards, revealing that the hands could belong either to one person, or to two people. Next, participants saw movies showing an interaction between the hands. In all conditions, one of the hands collected three blocks, and arranged them in a line. Subsequently, in the “contrary goals” condition, infants were shown events designed to provide them with clear evidence of a conflict between goals. As the first hand attempted to reach for a fourth block, the second hand intervened, preventing the first one from achieving its goal. This scenario leveraged infants’ demonstrated ability to process the goal of unfulfilled reaching gestures toward objects (Brandone & Wellman, 2009; Hamlin et al., 2007).

By contrast, the baseline condition was designed to minimize evidence of a thwarted goal while preserving the movement patterns of the contrary goals condition. In the baseline condition, participants saw the same videos as in the contrary goals condition, but we edited out the fourth block. Thus, in the baseline condition, the first hand’s goal of collecting objects was not thwarted when the second hand blocked the first one. As a result, the events shown in the baseline condition provided infants with much less evidence for a conflict between goals than the contrary goals condition.

Afterwards, during the test phase, the same outcome events were shown in all conditions. Participants saw that the hands belonged either to one person or to two different people. We anticipated that in the baseline condition, infants would look longer at the two people than to the one-person test outcome, simply because the two-people outcome would show more things on the screen (Bonatti et al., 2002; Futó et al., 2010; Xu & Carey, 1996; Xu et al., 2004). In the contrary goals condition, we anticipated that infants would assume that an agent is unlikely to prevent themselves from achieving their goals, thereby enhancing their assumption that two people might be found behind the screen. Therefore, in the contrary goals condition, we expected that infants’ tendency to look more at the two people than at the one-person outcome would be reduced (or entirely flipped) in comparison to the baseline condition.

### Methods

#### Participants.

Two groups of 16 9-month-old infants participated in Study 1A (Contrary goals condition: *M*_*age*_ = 271 days, *range* = 256–284 days, *SD* = 7.83 days; Baseline condition: *M*_*age*_ = 271 days, *range* = 262–287 days, *SD* = 6.79 days). Nine additional participants were excluded from data analysis because of a technical failure (3), not looking at a critical event required to process the test movies—see the Coding and Analysis section below (2), parental interference (2), fussiness (1), and reaching the maximum cumulative looks criteria in all the test trials (1). Sample sizes were modeled after comparable studies in which infants had to represent the goal of an approach toward one of two types of objects (Hernik & Southgate, 2012; Woodward, 1998). A sensitivity power analysis performed with G*Power (Faul et al., 2007) revealed that sample size was sufficient to detect medium to large effect sizes (f > .18) when assessing interactions between condition and type of test with an ANOVA (*α* = 0.05, power = .8, observed correlation between repeated measures = .75).

#### Apparatus.

Infants were tested in a dimly lit soundproof room in which they were seated on their caregiver’s lap 100 cm from a 40-in. LCD monitor on which the stimuli were presented. The caregivers were instructed to close their eyes during the procedure. A hidden camera (temporal resolution = 25 frames per second) recorded the infants’ looking behavior.

#### Stimuli and Procedure.

Each infant watched four familiarization trials, followed by four test trials. In between trials, a looming figure was presented at the center of the screen to attract participants’ attention.

#### Contrary Goals Condition.

##### Familiarization Phase.

**Figure 1.**
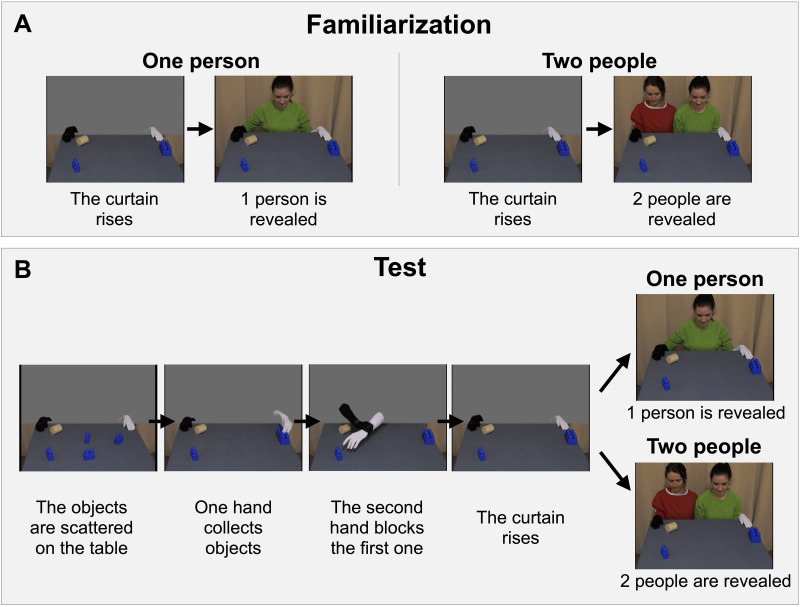
*Key Events from the Contrary Goals Condition of Study 1A*. *Note*. Panel A: Events shown during the familiarization phase. Hands are resting on the table and the curtain rises, revealing either one person or two people. Panel B: Interactions and outcomes shown during test trials. First, the white hand collects objects, before being blocked by the second hand and the grey screen slides upwards. Either one person or two people are revealed behind the screen at the end of test trials.

The experiment started with a familiarization phase in which infants were given evidence on the number of individuals that could be present in the videos (in two trials, one person; in two trials, two people; counterbalanced order). During each familiarization movie, participants first saw a still image of a table covered in grey cloth on which a wooden cylinder was placed on one side, and three identical Duplo blocks on the other side. A fourth identical Duplo block was placed on the side of the wooden cylinder, next to the edge of the table closest to the participants. Two hands wearing gloves (one white and one black) rested at the left and right upper end of the table. A grey screen covered the upper part of the scene, making it impossible to see to whom the hands belonged (Figure 1A). After 1 s, the grey screen disappeared by sliding upwards, revealing that the gloved hands belonged to either one person (*One person* trial, see Supplemental Material Video S1) or two people (*Two people* trial, see Supplemental Material Video S2). Then the movie froze for 2 s. The familiarization phase was followed by a presentation of four test trials.

##### Test Phase.

The two types of test trials were identical till their last scenes. Each test trial started with a movie showing an interaction between the two gloved hands (Figure 1B). At the beginning of these movies, the gloved hands, the wood cylinder, and one of the Duplo blocks occupied the same position as in the familiarization movies. The three other Duplo blocks were scattered on the table. A grey screen covered the upper half of the scene, preventing infants from seeing to whom the gloved hands belonged. First, the hand wearing the white glove collected these three blocks one by one and arranged them in a row (in the position that they occupied during the familiarization trials). This part of the movie was designed to help infants attribute the goal of collecting blocks to the first hand. This objective was achieved through several key features: the hand gathered several blocks, using varied trajectories to show equifinality, and arranged them in a line, thus yielding a noticeable effect. These cues—repetition, equifinality, and noticeable effect—are known to support infants’ goal encoding (e.g., Biro & Leslie, 2007; Hernik & Southgate, 2012; Woodward, 1998).

After collecting three blocks, the white-gloved hand reached for the fourth Duplo block. It was stopped mid-air by the black-gloved hand that grabbed the white-gloved hand’s wrist, thus preventing it from reaching the fourth Duplo block. During that time, the white-gloved hand moved its fingers towards the fourth Duplo block, as if trying to reach it. Next, the black-gloved hand released its grip on the white-gloved hand, and the hands moved back to their initial position on the table.

In each test trial, the movies showing an interaction between the hands were directly followed by an outcome movie, in which the grey screen disappeared by sliding upwards, revealing a still image showing how many people were manipulating the gloves (see Supplemental Material Video S3, Figure 1B). In two test trials, one person was revealed (*One person* trials). In the other two test trials, two people were revealed (*Two people* trials), order counterbalanced. The infants’ looking time at the screen was measured from the moment the grey screen fully disappeared until they looked away from the monitor for 2 s or more, or after 30 s had elapsed.

##### Counterbalanced Factors.

During the familiarization and test phases of Study 1A, each participant saw two one-person outcomes and two two-people outcomes, in one of the following four presentation orders (1/4^th^ of the participants per order): 1221; 1212; 2121; and 2112, where 1 stands for one-person outcomes, and 2 for two-people outcomes.

Two color sets and spatial layouts were used to minimize carry-over effects and boredom. One color set featured blue blocks, a beige curtain, one actress in green, and another in red. The other color set included red blocks, a green curtain, one actress in pink, and another in blue. In the first spatial layout, the black-gloved hand was on the screen’s left side, while the white-gloved hand was on the screen’s right side. A wood cylinder was placed next to the black-gloved hand, with three Duplo blocks on the right side and an isolated block in the bottom left of the screen. The second layout inverted the positions of objects, gloved hands, and people, following a central vertical axial symmetry. Each participant saw one trial of each color and spatial layout combination during familiarization and test. The order of presentation of color sets, the order of presentation of spatial layouts, and the pairing of color, layout, and trial type (one person vs. two people) were counterbalanced across participants.

For all studies reported in this paper, designs were fully balanced across all counterbalanced factors.

#### Baseline Condition.

In the baseline condition, the movies and procedure were the same as in the contrary goals condition, except that the fourth Duplo block toward which the hand reached when it was prevented by the other hand was digitally edited out (Supplemental Material Video S4). As a result, there was no evidence that the white-gloved hand’s reaching gesture was directed toward a specific goal object. Therefore, even if the hands were performing a strange action (one hand holding the other while the other one was extending its fingers), the baseline condition provided infants with less evidence of conflict between goals than the contrary goals condition.

Note that any end state can be a goal, such as, for instance, reaching towards an empty space. Thus, in principle, an adult observer might construe the baseline condition videos of Study 1A as providing evidence for a conflict between goals, with the first hand attempting to simply reach towards an empty space, whilst being blocked by the second hand. Our experiment was designed to prevent infants from forming such an interpretation. First, infants do not typically view reaching toward empty spaces as goal-directed, even when these behaviors are shown repeatedly (Phillips & Wellman, 2005). Second, in the baseline condition videos, the blocked hand reached only once toward an empty space, following a single path, and without producing any noticeable outcome. Thus, the reaching gesture directed towards an empty space shown in the baseline videos lacked the key features of repetition, equifinality, and noticeable effects that infants use to identify goals.

#### Coding and Analysis.

We analyzed infants’ looking behavior frame-by-frame during the measurement period of the test phase, coding looks at the screen versus looks away. Blinks lasting over 0.2 seconds were counted as looks away. Looking time was measured from when the movie froze, after the grey screen fully slid upward. The first author, aware of condition information, coded all data, and a second coder, blind to hypotheses, independently coded 50% of the recordings. Inter-rater agreement was high (average *r* = 0.98, range 0.96–1). When the difference between the values from the first and the second coder exceeded 10% of the value from the first coder, the discrepancy was resolved by discussion. Infants had to look at the screen when the black-gloved hand grasped the white-gloved hand’s wrist (revealing conflicting goals) to be included; two infants who did not meet this criterion were excluded. Looking times were then averaged across trials for each condition.

All statistical tests used in this paper were two-tailed and conducted with R (v.4.1.0) and R studio (v.1.4.1717), using the following packages: ggpubr (v.0.4.0), ggsci (v.2.9), dplyr (v.1.1.0), rstatix (v.0.7.0), afex (v.1.0.1), exact2x2 (v.1.6.8), and tidyr (v.1.3.0). Analyses of looking times were performed on log-transformed data (Csibra et al., 2016). For ease of reading, we always report untransformed looking times in the Figures and main text. Raw data and analysis scripts are available in the Supplementary Materials.

### Results and Discussion

**Figure 2.**
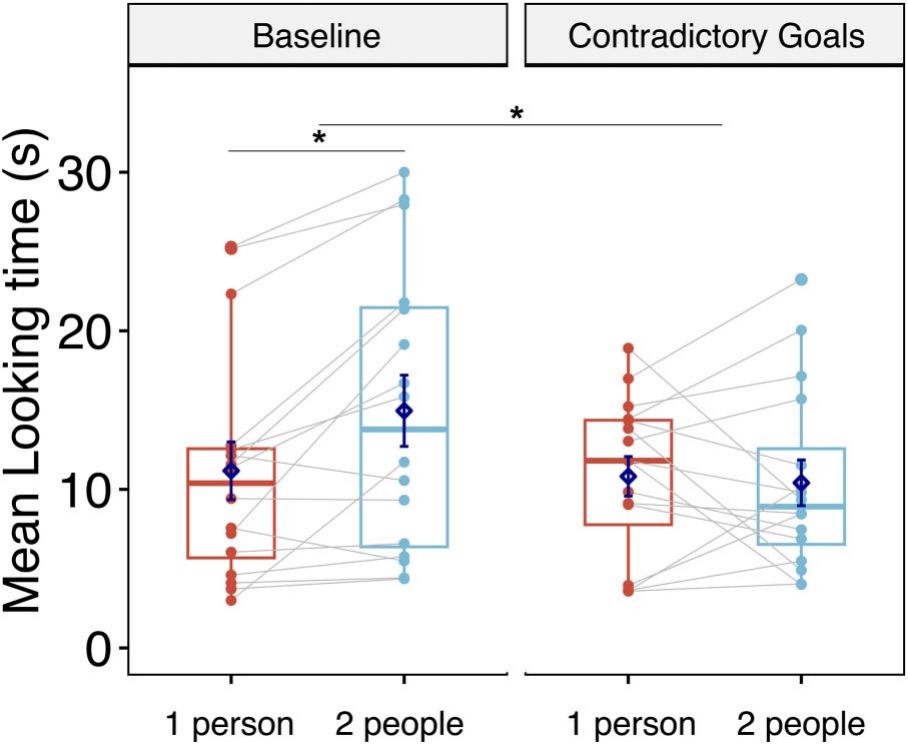
Average Looking Times to Study 1A’s Test Events per Condition (Baseline vs. Contrary goals) and Test Event (One person vs. Two people). *Note*. Dots represent individual data points; grey lines connect repeated measures from each individual. Diamonds represent means. Horizontal bars within boxes represent medians, and boxes indicate the middle 2 quartiles of data. Whiskers indicate data up to 1.5 times the interquartile range above the third quartile (upper whiskers), and up to 1.5 times the interquartile range below the first quartile (lower whiskers). *p < .05.

The results of Study 1A are shown in Figure 2. First, we ran a two-way ANOVA on log-transformed looking times during the test with the number of people (two vs. one) as a within-subject factor, and condition (contrary goals vs. baseline) and test order (1221, 1212, 2121, or 2112) as between-subject factors. The ANOVA revealed a marginally significant main effect of the number of people (*F*(1, 24) = 4.00, *p* = .057, *η*_*p*_^2^ = 0.14) and a significant interaction between condition and number of people (*F*(1, 24) = 4.53, *p* = .044, *η*_*p*_^2^ = 0.16, 95% CI [0, 0.39]). The ANOVA revealed no other significant effects. In short, the interaction suggests that the effect of the number of people present in the outcome differed across conditions.

Planned comparisons indicated that infants looked longer at the test outcome showing two people rather than one person in the baseline condition (14.95 vs. 11.17 s; *t*(15) = −3.14, *p* = .007, *d* = −0.78, 95% CI = [−1.3, −.50]). By contrast, in the contrary goals condition, infants did not look significantly longer at two people rather than at one person (10.41 vs. 10.82 s; *t*(15) = 0.08, *p* = .937, *d* = 0.02, 95% CI = [−.57, .52]).

In the baseline condition, infants looked longer at the two-people than at the one-person test outcomes. This result is not surprising given that in the two-people outcome, more visually interesting elements—faces and bodies in this study—were on the screen (for comparable results, see (Bonatti et al., 2002; Xu & Carey, 1996; Xu et al., 2004). Crucially, it was sufficient to provide unambiguous evidence for conflicting goals in the contrary goals condition to significantly disrupt infants’ baseline tendency to look longer at the two-people outcomes, as suggested by the interaction between number of people and condition. This result suggests that infants’ assumption that two people were manipulating the gloves increased because of their observation of one hand preventing the other from achieving its goals.

Yet, in Study 1A’s critical contrary goals condition, infants did not look longer when the test outcome showed one person rather than two people, presumably because of infants’ tendency to look longer at 2 people than at one person. Study 1B was designed to address this issue, by replicating conceptually Study 1A, while circumventing infants’ tendency to look longer at two people than at one person.

Study 1B was designed after Study 1A. However, it used a within subject design, displaying only one person outcomes at test. In Study 1B, 9-month-old infants were shown that one person operated the gloves after receiving either evidence for contrary goals (contrary goals condition), or after seeing the matched control event in which there was no target the hands might compete over (baseline condition). If infants assume that agents are unlikely to prevent themselves from achieving their goals, they should look longer at one person in the contrary goal condition than in the baseline condition. Unlike Studies 1A and 2 which were run when pre-registrations were still uncommon, Study 1B was pre-registered at https://osf.io/kb8de/?view_only=eeedb080e60a46c4ad93469f8e98c8d1.

## STUDY 1B

### Methods

#### Participants.

Thirty-two 9-month-old infants participated in Study 1B (*M*_*age*_ = 271 days, *range* = 256–284 days, *SD* = 7.83 days). Seventeen additional participants were excluded from data analysis because of a technical failure (3), experimental error (6), fussiness (7), and because the infant’s face was not visible, making it impossible to code data (1). An a priori power analysis conducted with G*Power indicated that a sample size of 32 participants was required to achieve a power of .95 for comparisons against chance by *t*-test for matched pairs, with *α* = .05, and assuming an effect size equal to .667 (i.e., the average effect size observed in looking time studies with infants, cf. Csibra et al., 2016).

#### Apparatus.

Infants sat approximately 60 cm from a 24-in. LCD monitor on which the stimuli were presented. Otherwise, Study 1B’s apparatus was identical to that of Study 1A.

#### Stimuli and Procedure.

Each infant watched the following sequence of trials: four familiarization trials, a first test trial, four reminder trials, and a second test trial. Between trials, a looming figure was presented at the center of the screen to attract participants’ attention.

##### Familiarization Trials.

**Figure 3.**
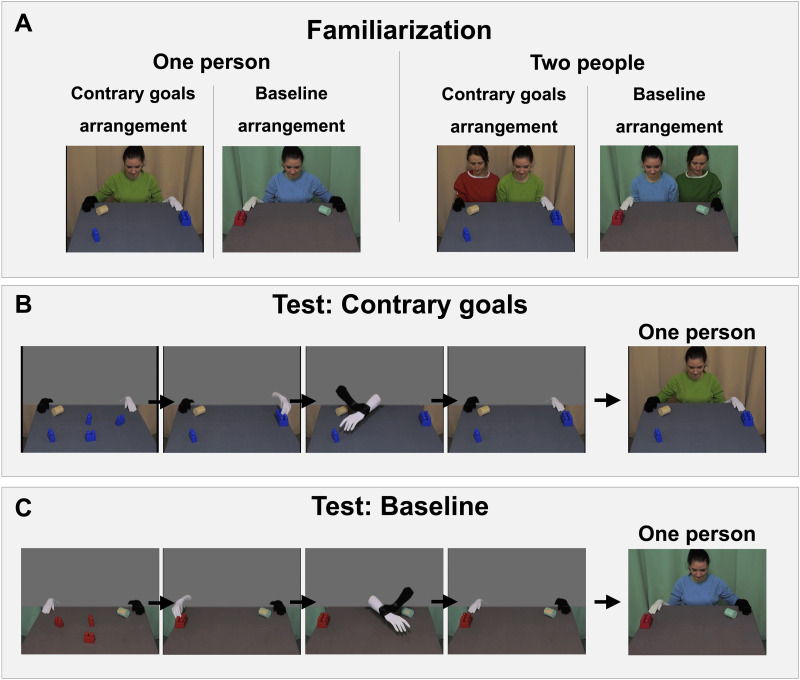
*Key Events from Study 1B*. *Note*. Panel A: Events shown during the familiarization phase. Hands are resting on the table and the curtain rises, revealing either one person or two people. In half of the familiarization movies, a fourth Duplo block is placed on the lower part of the table (contrary goals arrangement, matching the outcome of the contrary goals test trials), while it is absent in the other half (baseline arrangement, matching the outcome of the baseline test trials). Panel B: Test trials in the contrary goals condition. The white hand collects objects until blocked by the second hand when reaching for another object. The grey screen then slides up, revealing one person. Panel C: Test trials in the baseline condition. The white hand collects objects until blocked by the second hand when reaching toward empty space. The grey screen then slides up, revealing one person.

Study 1B started with four familiarization trials, which were identical to those used in Study 1A, with the following changes. First, Study 1B used within-subjects conditions. Thus, each participant saw two familiarization trials from the contrary goals condition, and two familiarization trials from the baseline condition. As in Study 1A, each familiarization trial began with a still image of a table covered in a grey cloth, which displayed a wooden cylinder on one side and three duplo blocks on the other. In the trials from the contrary goals condition, a fourth identical Duplo block was placed next to the wooden cylinder, while in trials from the baseline condition, this block was digitally edited out (Figure 3A). At the upper ends of the table, two gloved hands—one white and one black—were positioned. A grey screen initially covered the upper part of the scene. After one second, this screen slid upwards to reveal either one person or two people, at which point the image froze. Unlike in Study 1A, the movie’s last image remained on the screen until the participant looked away from the monitor for 2 s or more, or after 30 s elapsed.

For each condition, one familiarization trial showed two people, the other familiarization trial showed one person. Familiarization trials alternated conditions (contrary goals = A, baseline = B) in A-B-A-B or B-A-B-A order, counterbalanced across participants. The number of people revealed behind the screen during familiarization trials (one person = 1, two people = 2) was presented in 1-2-2-1 or 2-1-1-2 order, also counterbalanced across participants. The familiarization phase was followed by a presentation of two test trials, interspersed with reminder movies.

##### Test Trials.

Each test trial started with showing infants a movie showing an interaction between the hands, shown twice in a row. These movies were identical to those used in Study 1A. They showed the white glove collecting blocks, before being blocked by the black glove while moving towards a block (contrary goal condition, Figure 3B), or towards an empty location (baseline condition, Figure 3C). Once the movie showing an interaction between the hands had been presented twice, the grey screen disappeared by sliding upwards, revealing that one person was wearing the gloves. The movie then froze until infants looked away for 2 s or more, or until 30 s elapsed, marking the end of the trial. Thus, in Study 1B’s test phase, participants were always shown that a single person was behind the screen, unlike in Study 1A.

Each participant was shown two test trials: one in the contrary-goals condition, and the other in the baseline condition. The order of presentation of test trials (beginning with either contrary goals or baseline conditions) was counterbalanced across participants.

##### Reminder Trials.

Between the first and the second test trial, the participants were presented with four reminder trials to remind them that either one person or two people might operate the gloves. The videos and procedure of the reminder trials were identical to those of the familiarization trials, except that the last image of the reminder movies remained on screen for a fixed duration of 2 s. The number of people revealed behind the screen during reminder trials was presented using one the following orders (with one person = 1 and two people = 2): either 1-2-2-1 or 2-1-1-2, counterbalanced across participants.

##### Color Sets and Spatial Layouts.

Study 1B used the same color sets and spatial layouts as Study 1A (color set 1: blue blocks, beige curtain, one actress in green, the other in red; color set 2: red block, green curtain, one actress in pink, the other in blue; spatial layout 1: black gloved hand on the left side, white gloved hand on the right side; spatial layout 2: black gloved hand on the right side, white gloved hand on the left side). For each participant, the spatial layout and color set used in each condition were fixed, and they differed across conditions. The specific spatial layouts and color sets used for each condition were counterbalanced across participants. Reminder trials used the same color set and spatial layout as those used in the test trial that followed them.

#### Coding and Analysis.

During the familiarization trials, we coded infants’ looking times during the one-person familiarization trials. These measures served to assess infants’ initial tendency to look at the images used during the test phase (all one-person outcomes), prior to any exposure to gloves’ actions. We also coded infants’ looking times during test trials. In all cases, the infants’ looking time at the screen was measured from the moment the grey screen fully disappeared (revealing who operated the gloves) until infants looked away from the monitor for 2 s or more, or after 30 s elapsed.

Coding and double-coding procedures were the same as in Study 1A, except that the primary coder was blind to condition information. Inter-rater agreement was good (average *r* = 0.99, range = 0.95–1). As in Study 1A, analyses of looking times were performed on log-transformed data (Supplemental Material Table S1 reports the means for the untransformed and log-transformed data).

### Results and Discussion

**Figure 4.**
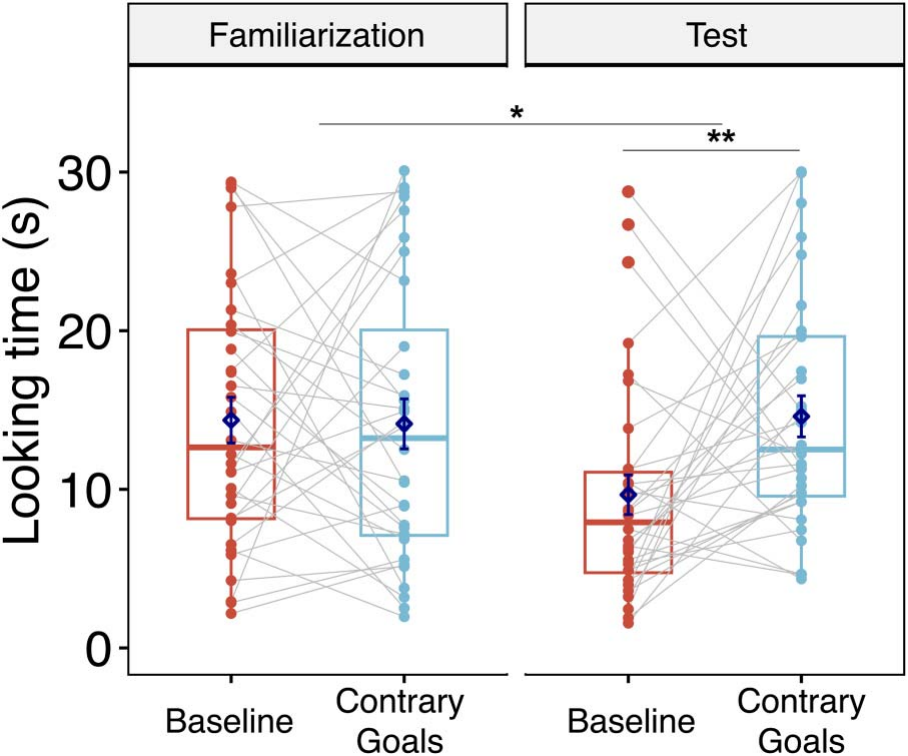
Looking Times to Study 1B’s One Person Outcome per Phase (Familiarization vs. Test) and Condition (Baseline vs. Contrary Goals). *Note*. Dots represent individual data points; grey lines connect repeated measures from each individual. Diamonds represent means. Horizontal bars within boxes represent medians, and boxes indicate the middle 2 quartiles of data. Whiskers indicate data up to 1.5 times the interquartile range above the third quartile (upper whiskers), and up to 1.5 times the interquartile range below the first quartile (lower whiskers). *p < .05.

The results of Study 1B are shown in Figure 4. To analyze Study 1B’s data we ran a full-factorial ANOVA on log-transformed looking times at outcomes showing that one person was behind the screen. Phase (familiarization vs. test), and condition (baseline vs. contrary goals) entered this analysis as within-subject factors, while test order did so as a between-subject factor (contrary goals condition first vs. baseline condition first). The ANOVA revealed a statistically significant main effect of condition (*F*_1, 30_ = 4.94, *p* = .034, *η*_*p*_^2^ = 0.14, 95% CI [0, 0.36]), indicating that participants’ average looking time was higher in the contrary goals condition (*M* = 14.60, *SD* = 7.33), than in the baseline condition (*M* = 9.66, *SD* = 7.03). Thus, in line with our hypotheses, participants looked significantly longer when discovering that a single person operated the gloves after receiving evidence for conflicting goals than in a matched baseline condition. Moreover, the ANOVA revealed a statistically significant interaction between condition and test phase (*F*_1, 30_ = 6.34, *p* = .017, *η*_*p*_^2^ = 0.17, 95% CI [0, 0.39]). This result indicates that the effect of condition differed across phase (familiarization vs. test). We investigate this difference in planned follow-up analyses below.

The ANOVA also showed a statistically significant interaction between condition and test order (*F*_1, 30_ = 5.03, *p* = .032, *η*_*p*_^2^ = 0.14, 95% CI [0, 0.36]). Thus, in Study 1B, participants looked longer at test outcomes in the contrary goals condition than in the baseline condition only when contrary goals trials appeared first. When they were presented second, prior exposure to a single person operating the gloves in baseline trials might have reduced participants’ surprise at discovering a single person behind the screen in the contrary goals condition.

Planned follow-up *t*-tests for matched pairs indicated that condition had no detectable effect on participants’ average looking times during the familiarization phase (contrary goals condition: *M* = 14.10, *SD* = 8.92 vs. baseline condition: *M* = 14.40, *SD* = 8.15, *t*(31) = .34, *p* = .732, *d* = 0.06, 95% CI = [−0.29, 0.46]). By contrast, during the test phase, participants’ average looking times were significantly higher in the contrary goals condition (*M* = 14.60, *SD* = 7.33), than in the baseline condition (*M* = 9.66, *SD* = 7.03, *t*(31) = −3.19, *p* = .003, *d* = −.56, 95% CI = [−0.95, −0.23]). In short, the effect of condition was specifically observed during the test phase, after observing interactions between hands.

In sum, in Study 1B’s test trials, two hands interacted before test outcomes revealed that they belonged to a single person. Participants looked longer at test outcomes if the hands’ interaction showed clear evidence for contrary goals (in the presence of a target object), than if the hands engaged in a matched control interaction (in the absence of a target object). Thus, infants perceived the presence of a single actor as less expected in the context of contrary goals than in the baseline condition. Notably, the difference in looking time at one person following the contrary goals vs. the baseline display was not significant in Study 1A, potentially due to limited statistical power for a between-subjects comparison (in contrast to the within-subjects design employed in Study 1B).

Together, Studies 1A and 1B suggest that infants apply assumptions of coherence over actions directed towards the achievement of simple physical goals. Study 2 investigated whether infants’ assumptions of coherence extend to communicative actions.

## ASSUMPTIONS ABOUT THE COHERENCE OF COMMUNICATIVE ACTIONS

The assumption that individuals pursue their communicative goals with some degree of rationality is likely to support the interpretation of speakers’ meanings (Grice, 1989; Sperber & Wilson, 1986). Thus, some assumption of coherence is likely to guide the interpretation of communicative actions. Just like assumptions of coherence operating over the interpretation of other actions, assumptions of communicative coherence need not be unlimited. Indeed, people sometimes contradict themselves, by mistake, or when they lie. Yet, one might assume that communicating individuals tend to be generally consistent. When one communicates, one aims for achieving some effects on someone’s mind. Thus, it is unlikely that a communicating individual would aim for producing an effect and its exact opposite—such as aiming for someone to believe p and not-p at the same time. Moreover, even when lying, individuals strive to appear consistent—if anything to avoid getting caught and to bolster their reputation (Mercier, 2012). Thus, a single speaker is unlikely to maintain blatant contradictions—whereas two speakers might very well keep disagreeing openly.

Adults spontaneously factor this type of coherence assumption in their processing of speakers’ meanings. Imagine Mary claiming “The cat is outside”, right before John interjects: “No! The cat is inside”. In this case, an observer might assume that Mary and John disagree—presumably because they hold contradictory beliefs about the cat’s location. Now consider the exact same sequence of utterances, but assume that they are produced by Mary alone. First, she claims “The cat is outside”, right before saying: “No! The cat is inside”. In that case an observer is unlikely to assume that Mary claims that the cat is both inside and outside at the same time. Instead, the interpretation of Mary’s utterances is shaped by the assumption that she is unlikely to contradict herself. For instance, an observer might rationalize Mary’s behavior by assuming that she revised her belief about the cat’s location—she claims that the cat is inside, and no longer claims that it is outside. In Study 2, we assessed whether infants rely on comparable assumptions of coherence when interpreting communicative actions.

## STUDY 2

### Operationalization Principle

Study 2 tested whether infants rely on assumptions of coherence when processing what people communicate. We focused on infants’ interpretation of a familiar communicative action (pointing), and tested 15-month-old infants because by that age, their capacity to follow pointing to retrieve objects is well-established (Behne et al., 2005, 2012; Mascaro & Kovács, 2022). The participants were enrolled in a hiding game in which they had to discover the location of a toy hidden in one of two containers (A and B). During familiarization trials, two informants took turns to indicate (truthfully) the location of the toy by pointing. Next, the participants were enrolled in two types of test trials. In the “one informant” test trials, a single informant pointed towards A and said “It’s there!”. Subsequently, she uttered an exclamation typically used to convey surprise (“Oh!”), and pointed towards B while saying “It’s there!” again. To describe it with an intentional gloss, the scene was designed to convey that the informant noticed that she made a mistake, and corrected her past communicative action by pointing a second time. We anticipated that if infants apply an assumption of coherence over communicative actions, they would not assume that the informant would claim that the same toy is both in A and B—a physical impossibility. Instead, they would assume that when pointing a second time, the informant no longer claimed that the toy was in A, and claimed that it was in B instead. Thus, we anticipated that infants would follow the second pointing in the one informant test trial. The two informants test trial was identical to the one informant test trial, except that the two pointing gestures were produced by two distinct informants. In that case, we assumed that infants would assume that the two informants disagreed on the location of the toy. Given that the two informants were equally familiar and reliable in the past, infants had no reason to trust one more than the other. Thus, we anticipated that in the two informants test trial, infants would follow both pointing gestures equally.

### Methods

#### Participants.

Forty 15-month-old infants participated in Study 2 (*M*_*age*_ = 469 days, *range* = 445–485 days, *SD* = 8.65 days). Additional participants were excluded from data analysis because of refusing to search inside the boxes (5), running to the boxes before the experiment could point (2), experimental error (1), technical failure (1), parental interference (1), and failing to understand the game, i.e., not finding the toy following pointing in more than half of the familiarization trials (11). A sensitivity power analysis performed with G*Power (Faul et al., 2007) for within-subject comparisons across types of tests with McNemar tests (*α* = 0.05, power = .8, proportion of discordant pairs = .5), indicated that our sample size was sufficient to detect medium to large effects—i.e., with odds ratios > 3.96 (Chen et al., 2010). The study was approved by an independent ethical review committee (Hungarian Ethical Review Committee for Research in Psychology, EPKEB; code: 2013/39), and the parents of all participants gave their written informed consent before inclusion.

#### Apparatus.

Infants were tested in a quiet soundproofed room. Two cameras (temporal resolution = 25 frames per second) recorded the infants’ behaviors.

#### Stimuli and Procedure.

The participants had to find a plush toy hidden in one of two red opaque plastic boxes (30x23x42 cm). At the beginning of each trial, participants were standing on the floor and were held by their caregiver or sitting on her lap. The caregiver sat on a beanbag. Two experimenters were standing in front of the participants, approximately 3.5 meters away from them. The boxes were placed centrally close to each other, approximately 2.5 meters away from the infant and their openings were oriented towards the experimenters. Thus, from their viewpoint, the participants could not see what was inside the boxes. The participants were enrolled in familiarization and test trials in the following order: 4 familiarization trials – first test trial – 2 familiarization trials – second test trial. The procedures of familiarization and test trials are shown in Supplemental Material Video S5.

##### Familiarization Trials.

At the beginning of each familiarization trial, one of the experimenters hid the toy that the participants had to find. First, she knelt down behind the boxes and showed the toy to the participant by holding it in her two hands above the boxes while saying « *Look here!* ». Second, the experimenter lowered the toy with two hands at the midline of the boxes. Once the toy was no longer visible to the participant, the experimenter placed it inside one of the boxes. She also simultaneously placed her remaining empty hand in the other box—so that the infant could not use information about the experimenter’s hiding movements to determine in which box the toy was placed. Next, the experimenter moved the two boxes approximately 1.2 m apart symmetrically from the midline and she stood up before going back to the standing position that she occupied at the beginning of the trial.

Next, one of the experimenters pointed towards the baited box. She ensured that the participant was looking at her by saying: « *Look!* » (throughout the procedure, experimenters raised their eyebrows and made eye contact with the participants when they talked to them). Then E1 pointed by fully extending her arm and index finger towards the baited box while turning her head towards it, and saying « *It’s there! Come and find it! *. » The sentence « *Find it!* » was the cue for caregivers to let participants approach the boxes.

The experimenters waited until the participant retrieved the toy, with their gaze oriented downwards, without looking at the boxes or at the child. If participants did not select any box or did not find the toy after a delay of approximately 30 s, the experimenter who pointed during the trial pointed once more at the baited box while saying: “*See, it’s here*”, before handing the toy to the participant. In all cases, after participants retrieved the toy, the experimenters congratulated them by clapping and excitedly saying “*Well done!*” before proceeding to the next trial. In order to be included in data analysis, participants had to follow the experimenter’s pointing gesture in at least 4 out of 6 familiarization trials.

##### Test Trials.

During the test phase, the participants were enrolled in one “one informant” test trial and in one “two informants” test trial, interspersed with two familiarization trials. Each test trial unfolded just like the familiarization trials, with the following exception. In the two informants test trial, the two experimenters pointed successively to provide contradictory information about the location of the toy. A first experimenter pointed to one of the boxes for about two seconds, while saying “*It’s here!*”. After the first informant stopped pointing, the second experimenter pointed towards the other box for about two seconds while saying: “*Oh! It’s here!*”. The one informant test trial was identical to the two informants test trial, except that the same experimenter produced the two consecutive contradictory communicative actions. Thus, in the one informant test trial, one experimenter pointed towards one of the boxes while saying “*It’s here!*” before pointing towards the other box while saying “*Oh! it’s here!*”. During all test trials, the baited box was always the one to which the experimenters pointed second. The test trials ended after infants retrieved a toy. If the participants did not retrieve any toy after 30 seconds elapsed from the end of the last pointing gesture, the infant was led back to her parent, and the test trial was repeated. Parents were instructed to close their eyes during the test trials.

##### Counterbalanced Factors.

For familiarization trials the following factors were counterbalanced across trials: the side of the baited box (right vs. left); the identity of the experimenter who hid the toy (experimenter 1 vs. 2); and whether the experimenter pointing was also the one who hid the toy (yes vs. no).

For test trials, the following factors were counterbalanced across participants: the identity of the first pointer (experimenter 1 vs. 2); in the one informant condition, whether the person who hid the toy was the person pointing (yes vs. no); in the two informants condition, whether the person who hid the toy was the person pointing first (yes vs. no).; the presentation order of test trials (one informant vs. two informants first), and the side of the baited box (right vs. left). The baited box was always the one that was pointed at second during test trials.

#### Coding and Analysis.

For each trial, we considered that participants followed a pointing gesture if they explored first the box that this pointing gesture was directed towards. The first author (aware of condition information) coded the data. Fifty percent of the data was also randomly selected and coded again by a second coder unaware of the hypotheses of the study (familiarization trials: average *kappa* = .91, range = .73 to 1; test trials: average *kappa* = 1). Disagreements between coders were resolved by discussion. Data analysis was conducted on the data from the first coder, except for trials in which there was a disagreement between coders. In these cases, the coding that the two coders agreed upon after discussion was kept in the analysis.

### Results and Discussion

**Figure 5.**
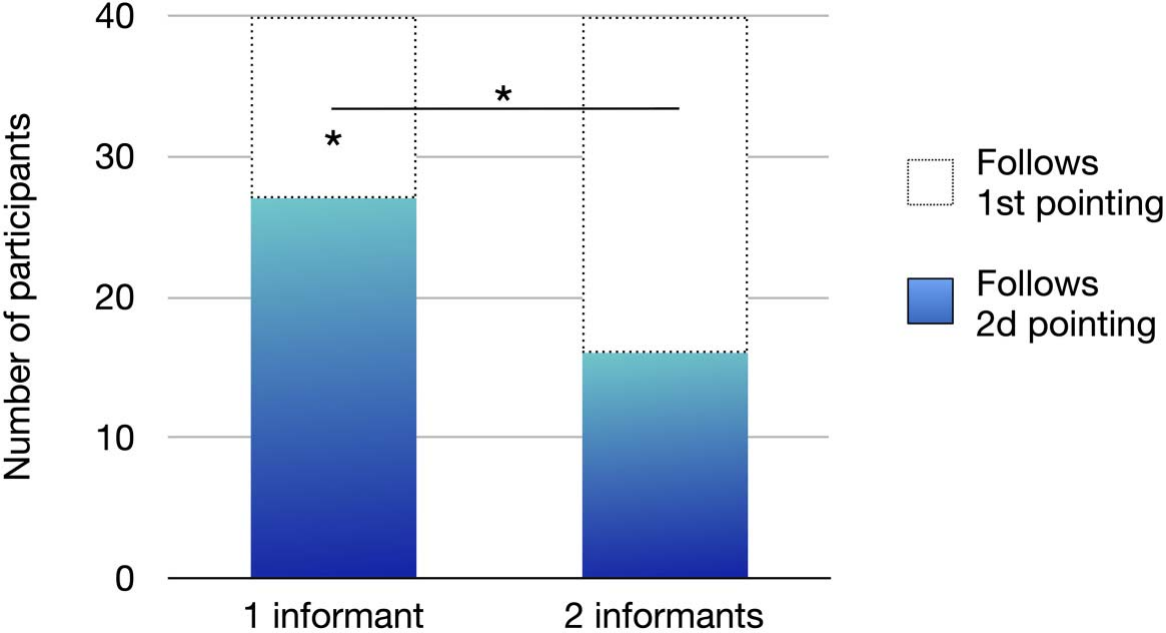
Number of Participants Following Each Pointing Gesture (First vs. Second) per Condition (1 Informant vs. 2 Informants). *Note*. *p < .05.

The results of Study 2 are shown in Figure 5. Infants followed the second pointing more often than predicted by chance in only in the one informant condition (27 infants/40, proportion = .675, 95% CI = [.51, .81], *p* = .038, binomial test), and not in the two informants condition (16 infants/40, proportion = .4, 95% CI = [.25, .57], *p* = .268, binomial test). Moreover, 18 out of 40 infants changed their strategy in the predicted direction. Specifically, they followed the first pointing in the two informants condition, and the second pointing in the one informant condition (proportion = .45, 95% CI = [.29, .61], *p* = .006, four-choice binomial test). To assess whether this pattern differed from chance, we used a four-choice binomial test because participants completed two test trials, each with two possible responses. Thus, they had a 1-in-2 chance of following the first pointing in the two informants condition, and a 1-in-2 chance of following the second pointing in the one informant condition, resulting in a 1-in-4 chance of displaying both behaviours by chance.

Seven infants changed their strategy in the opposite direction, following the second pointing in the two informants condition, and the first pointing in the one informant condition (proportion = .17, 95% CI = [.07, .33], *p* = .361, four-choice binomial test). Infants’ changes of strategy were more likely to occur in the predicted direction than in the opposite direction (18 vs. 7, Exact McNemar test, *p* = .043, *OR* = .38, 95% CI = [.14, .98]).

The remaining 15 infants did not change their strategy across conditions. Of these, six always followed the first pointing (proportion = .15, 95% CI = [.06, .30], *p* = .199, four-choice binomial test), and nine always followed the second pointing (proportion = .22, 95% CI = [.11, .38], *p* = .856, four-choice binomial test).

An additional exploratory analysis showed no evidence that infants trusted the experimenter who hid the toy more than the other experimenter in the two informants condition (16/40 infants followed the hider’s point, proportion = 0.4, 95% CI [0.25, 0.57], *p* = .268, binomial test). This pattern of results was expected, since both informants were equally familiar and reliable during familiarization, and both were present and attentive during the hiding phase.

## GENERAL DISCUSSION

Overall, our results suggest that infants apply assumptions of coherence over reaching (a simple instrumental action), and pointing (a communicative action). Studies 1A and 1B revealed that 9-month-old infants can use their sensitivity to how constraints hinder goal achievement to draw inferences that are consistent with a psychological coherence principle. Their assumptions that two different individuals might interact was stronger when they received evidence of conflicting goals than when they saw the same behaviors, but the conflict between goals was less apparent, as the goal object was absent. Therefore, infants recognized that one person was unlikely to prevent herself from achieving her goals. In Studies 1A and 1B, infants might have been sensitive to contrariety at two different levels. First, they might have assumed that one agent is unlikely to try to achieve an end state (e.g., “p”) and an incompatible end state (e.g., “not-p”) at the same time. A second possibility is that infants assumed that an agent selected means to support the achievement of their goals rather than prevent them. Our data do not allow us to separate these interpretations. Nonetheless, all of them imply that infants are sensitive to psychological coherence.

Results from Studies 1A and 1B imply that infants are sensitive to the opposition relation between supporting and preventing the achievement of a goal. In principle, such a sensitivity can be implemented in one of two ways. The first possibility is that infants are sensitive to the opposition between enabling the achievement of a goal and preventing it, without representing the opposition relation itself (Bermúdez, 2006; Millikan, 2006). For example, the Necker cube (Necker, 1832) is perceived in only one of two ways at a time. This phenomenon is likely to reflect a competition process between two representations, which may be implemented by a relationship of mutual inhibition. However, it does not necessarily involve representing that the two different interpretations of the Necker cube are mutually incompatible.

Alternatively, infants might represent the relation of opposition between preventing and enabling the achievement of a goal. Infants’ inference about the number of agents based on the existence of conflicting goals evidenced in Studies 1A and 1B supports this second interpretation. Indeed, if infants were simply treating preventing and enabling the achievement of a goal as competing representations, without representing the opposition relation between them, they should not be able to draw inferences on the number of agents present after observing one hand preventing another from achieving its goal. Similarly, infants infer agents’ dominance status by tracking who prevails when agents’ goals are conflicting (Bas & Sebastian-Galles, 2021; Enright et al., 2017; Gazes et al., 2017; Mascaro & Csibra, 2012, 2014; Mascaro et al., 2023). In these studies, infants’ capacity to draw social inferences based on conflicting goals is difficult to understand unless infants represent the relation of opposition between contrary plans of actions.

Study 2’s results suggest that infants apply assumptions of coherence over communicative actions. In Study 2, informants pointed to indicate the location of a single toy, hidden in one of two boxes. When one informant pointed to A, and next, another informant pointed to B, infants were equally likely to follow either pointing. Conversely, when the two consecutive pointing gestures were produced by a single informant, infants followed the second pointing more often than the first one. Thus, infants did not assume that the single informant pointing twice maintained two contradictory assertions. Instead, they were sensitive to the fact that when the single informant pointed a second time, she cancelled her initial assertion. In Study 2, infants’ assumptions of communicative coherence is consistent with several non-mutually exclusive hypotheses. First, infants might form assumptions over the type of information that speakers convey. For instance, they might assume that speakers are unlikely to assert blatant contradictions. Second, infants might assume that people generally assert what they believe, and assume people’s sets of beliefs to be coherent. Third, infants might assume that when people communicate, they attempt to achieve certain effects on other people’s minds, with some degree of rationality. According to this hypothesis, communicating individuals would be unlikely to aim for both supporting and preventing the achievement of their communicative goals.

Here, we investigated how assumptions of psychological coherence play a role in the representation of instrumental and communicative actions. These assumptions are crucial to support representations of agency by guiding inferences about how multiple sets of plans are likely to be integrated. Assumptions of psychological coherence also contribute to setting the boundaries of the mind. However, the study of principles that organize sets of mental states within a mind should be extended to many more domains. The principle of psychological coherence predicts, for example, that a single mind is unlikely to believe “p,” and “not-p” at the same time. Therefore, the principle of psychological coherence should support the representation of inferences in others—just like the law of non-contradiction is a crucial underpinning of logical reasoning. Similarly, the principle of psychological coherence predicts that a single mind is unlikely to know and ignore something at the same time. Therefore, assumptions of psychological coherence should result in an assumption of transparency of the mind, such that a single mind cannot hide things from itself, whereas several different minds can hide information from each other. How the mechanisms supporting these assumptions of coherence operate and how they are implemented in human adults, infants, and non-human animals are important questions for future research.

## ACKNOWLEDGMENTS

We thank our participants and CEU’s Cognitive Development Center, especially Zs. Karap, B. Széplaki-Köllőd, and M. Tóth. We also thank Lindsey Powell and two anonymous reviewers for their constructive feedback on the manuscript.

## FUNDING INFORMATION

This research was funded by grants from the European Commission (REPCOLLAB, ERC-2011-StG_20101124), and from the Austrian Science Fund (FWF, DOI 10.55776/F100600) to Á.K. It also received support from grants from the « Agence Nationale pour la Recherche » (FoundTrust, ANR-21-CE28-0017-01; LUCID: ANR-25-CE28-5534) to O.M. This work contributes to the investment programme “France 2030” as part of the IdEx programme (ANR-18-IDEX-0001) implemented by Université Paris Cité, under which the inIdEx project EFL (Empirical Foundations of Linguistics) is conducted.

## AUTHOR CONTRIBUTIONS

O.M.: Conceptualization; Data curation; Formal analysis; Writing – original draft; Writing – review & editing. Á.K.: Conceptualization; Writing – original draft; Writing – review & editing.

## DATA AND MATERIALS AVAILABILITY STATEMENTS

Detailed descriptive statistics are reported in the article. Data and analysis scripts are available in the Supplementary Materials.

## Supplementary Material












